# Nomograms for assessing the rupture risk of anterior choroid artery aneurysms based on clinical, morphological, and hemodynamic features

**DOI:** 10.3389/fneur.2024.1304270

**Published:** 2024-02-08

**Authors:** Shijie Zhu, Xiaolong Xu, Rong Zou, Zhiwen Lu, Yazhou Yan, Siqi Li, Yina Wu, Jing Cai, Li Li, Jianping Xiang, Qinghai Huang

**Affiliations:** ^1^Department of Neurovascular Center, Changhai Hospital, Naval Medical University, Shanghai, China; ^2^ArteryFlow Technology Co., Ltd., Hangzhou, Zhejiang, China; ^3^Department of Neurosurgery, 971 Hospital of People's Liberation Army (PLA), Qingdao, China; ^4^Department of Neurosurgery, Linyi People's Hospital, Linyi, China; ^5^Cerebrovascular Department of Interventional Center, Henan Provincial People's Hospital, Zhengzhou, China

**Keywords:** anterior choroid artery aneurysm, hemodynamics, morphology, nomograms, rupture risk

## Abstract

**Background and purpose:**

A notable prevalence of subarachnoid hemorrhage is evident among patients with anterior choroidal artery aneurysms in clinical practice. To evaluate the risk of rupture in unruptured anterior choroidal artery aneurysms, we conducted a comprehensive analysis of risk factors and subsequently developed two nomograms.

**Methods:**

A total of 120 cases of anterior choroidal artery aneurysms (66 unruptured and 54 ruptured) from 4 medical institutions were assessed utilizing computational fluid dynamics (CFD) and digital subtraction angiography (DSA). The training set, consisting of 98 aneurysms from 3 hospitals, was established, with an additional 22 cases from the fourth hospital forming the external validation set. Statistical differences between the two data sets were thoroughly compared. The significance of 9 clinical baseline characteristics, 11 aneurysm morphology parameters, and 4 hemodynamic parameters concerning aneurysm rupture was evaluated within the training set. Candidate selection for constructing the nomogram models involved regression analysis and variance inflation factors. Discrimination, calibration, and clinical utility of the models in both training and validation sets were assessed using area under curves (AUC), calibration plots, and decision curve analysis (DCA). The DeLong test, net reclassification index (NRI), and integrated discrimination improvement (IDI) were employed to compare the effectiveness of classification across models.

**Results:**

Two nomogram models were ultimately constructed: model 1, incorporating clinical, morphological, and hemodynamic parameters (C + M + H), and model 2, relying primarily on clinical and morphological parameters (C + M). Multivariate analysis identified smoking, size ratio (SR), normalized wall shear stress (NWSS), and average oscillatory shear index (OSI_ave_) as optimal candidates for model development. In the training set, model 1 (C + M + H) achieved an AUC of 0.795 (95% CI: 0.706 ~ 0.884), demonstrating a sensitivity of 95.6% and a specificity of 54.7%. Model 2 (C + M) had an AUC of 0.706 (95% CI: 0.604 ~ 0.808), with corresponding sensitivity and specificity of 82.4 and 50.3%, respectively. Similarly, AUCs for models 1 and 2 in the external validation set were calculated to be 0.709 and 0.674, respectively. Calibration plots illustrated a consistent correlation between model evaluations and real-world observations in both sets. DCA demonstrated that the model incorporating hemodynamic parameters offered higher clinical benefits. In the training set, NRI (0.224, *p* = 0.007), IDI (0.585, *p* = 0.002), and DeLong test (change = 0.089, *p* = 0.008) were all significant. In the external validation set, NRI, IDI, and DeLong test statistics were 0.624 (*p* = 0.063), 0.572 (*p* = 0.044), and 0.035 (*p* = 0.047), respectively.

**Conclusion:**

Multidimensional nomograms have the potential to enhance risk assessment and patient-specific treatment of anterior choroidal artery aneurysms. Validated by an external cohort, the model incorporating clinical, morphological, and hemodynamic features may provide improved classification of rupture states.

## Introduction

The occurrence of anterior choroidal artery (AChA) aneurysms is relatively low, constituting only 2–5% of all cerebral aneurysms (1). While the incidence of rupture in AChA aneurysms is elevated within the realm of medical care (2), the PHASES score scale (considering population, hypertension, age, size, prior subarachnoid hemorrhage, and location) indicated relatively lower scores for these small aneurysms (3–5). Furthermore, earlier research showed that the majority of ruptured intracranial aneurysms (IAs) exhibit a diameter of less than 7 mm (6), with approximately 35–47% of all IAs being small and ruptured (7, 8). Given these findings, a conservative monitoring approach for predominantly small AChA aneurysms may potentially expose patients to aneurysmal subarachnoid hemorrhage (SAH). This may suggest that the simple, all-position assessment models do not apply to AChA aneurysms characterized by small size. Simultaneously, a precise evaluation of unruptured AChA aneurysms holds the potential for significant socioeconomic benefits, mitigating the costs and hazards associated with unnecessary interventions.

Moreover, both endovascular and surgical interventions for unruptured intracranial aneurysms (UIAs) entail a risk of post-procedural complications, encompassing ischemic events, hydrocephalus, and neurological deficits (9). Intensive therapy may not yield significant benefits for patients with unruptured IAs, especially those with a low probability of lesion rupture (10). Therefore, the timely identification of AChA aneurysms exhibiting an elevated risk of rupture, coupled with prompt intervention to forestall potential disastrous outcomes, assumes paramount importance in the realm of medical treatment.

To the best of our knowledge, AChA aneurysms have received limited consideration in existing aneurysm risk assessment models and scoring systems (11–16). Furthermore, there is a paucity of documented research on risk factors and assessment models specifically tailored for AChA aneurysm rupture. In recent years, nomograms have gained widespread acceptance as a predictive method for IAs (3, 13), meeting the criteria for integrated models and advancing personalized healthcare (17). In our pursuit of establishing patient-specific nomogram models for AChA aneurysms, our research group diligently explored rupture risk factors through the analysis of clinical, morphological, and hemodynamic parameters. Additionally, we sought to develop user-friendly and multidimensional nomograms that align with clinicians’ needs for high efficiency and accuracy in estimating the probability of AChA aneurysm rupture.

## Materials and methods

### Participants and study design

Our retrospective cohort study received approval from the ethical committee of the hospital; however, informed consent was waived. Before data collection, all patient information underwent anonymization.

**Figure 1:**
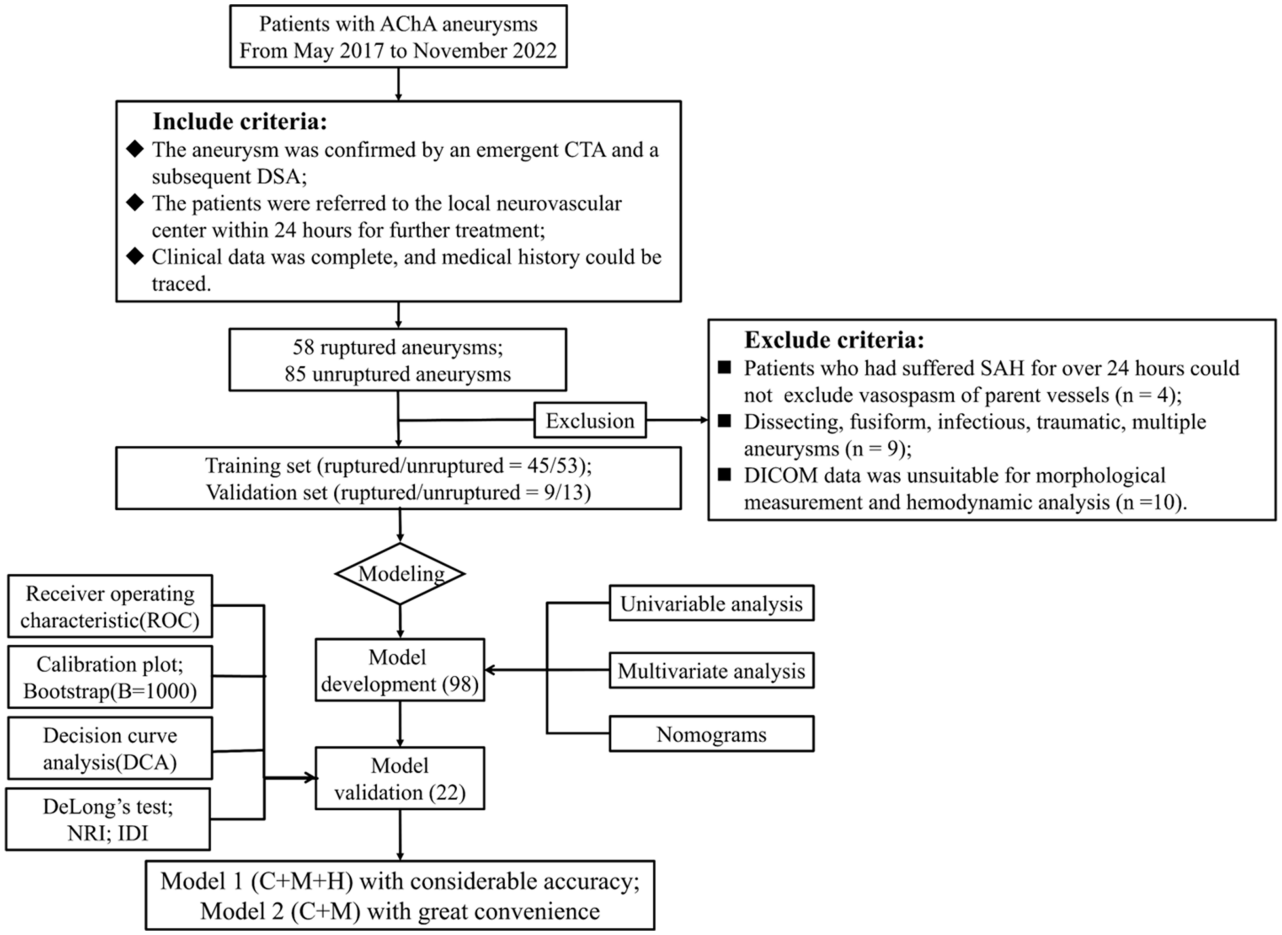
Flowchart of study design. AChA aneurysms enrolled from May 2017 to November 2022 established both the training and validation cohorts, which were adopted to develop the nomogram models; 98 and 22 cases were adopted for nomogram construction and external testing. Discrimination, calibration, and clinical utility were illustrated by ROC, calibration plots, and DCA, respectively. Moreover, we performed the DeLong test, NRI, and IDI to compare the performance of two nomogram models. AChA, anterior choroid artery; CTA, computed tomographic angiography; DSA, digital subtraction angiography; SAH, subarachnoid hemorrhage; DICOM, digital imaging and communications in medicine; NRI, net reclassification index; IDI, integrated discrimination improvement; C + M + H, clinical, morphological, and hemodynamic features; C + M, clinical and morphological features, SR, size ratio; NWSS, normalized wall shear stress; OSI_ave_, average oscillatory shear index.

We obtained cerebrovascular imaging and medical records of 120 consecutive patients with AChA aneurysms admitted to 4 medical units between May 2017 and November 2022. Based on their admission status, we classified the aneurysms as either ruptured or unruptured. Inclusion criteria were as follows: (1) patients diagnosed with an AChA aneurysm through emergency computer tomography angiography (CTA) followed by digital subtraction angiography (DSA); and (2) availability of complete clinical data and traceable medical history. Exclusion criteria included the following: (1) patients with subarachnoid hemorrhage (SAH) lasting over 24 h where vasospasm of parent vessels could not be ruled out; (2) individuals with dissecting, fusiform, infectious, traumatic, or multiple aneurysms; (3) patients whose Digital Imaging and Communications in Medicine (DICOM) data did not permit morphological measurements and hemodynamic calculations; and (4) AChA aneurysms with insufficient information and data. Ultimately, 98 AChA aneurysms (45 ruptured and 53 unruptured) from 3 hospitals contributed to the development of the nomograms, while an additional 22 cases (9 ruptured and 13 unruptured) from a fourth hospital were included for external validation. For more detailed information, refer to the flowchart (Figure 1).

### Acquisition, reconstruction, and analyses of AChA aneurysm images

User-friendliness hinges on a commitment to openness and transparency throughout the entire process. In the image acquisition phase, original DSA images are procured from the Artis zee biplane angiographic system (VC14, Siemens, Munich, Germany) as 490 files in DICOM format. The procedure using MIMICS 21.0 software (Materialise NV, Leuven, Belgium) unfolds as follows: Data are imported via “New Project,” settings are adjusted through “Threshold” until blood vessels are distinctly displayed, the region of interest is selected in “Crop Mask” for 3D image reconstruction, and the resultant file is exported in STereoLithography (STL) format. AneuFlow™ software (V1.1.4.1, ArteryFlow Technology, Hangzhou, China) for measuring aneurysm morphology and hemodynamics encompasses three steps (18). First, the STL file is imported into the AneuFlow™ software, allowing doctors to obtain additional morphological parameters by manually identifying the aneurysm neck. The vascular model is segmented into two regions: the parent artery and the aneurysm. Second, the computational fluid dynamics (CFD) simulation module initiates hemodynamic analysis. The maximum mesh size is set at 0.16 mm with three layers of wall prism elements. The aneurysm model comprises 1.8–2.3 million tetrahedral elements post-meshing (19). An implicit unsteady solver with a first-order upwind numerical scheme is employed. By resolving the Navier–Stokes governmental equations and assuming solid walls, AneuFlow™ software conducts laminar and incompressible blood flow simulations (density = 1,056 kg/m^3^ and viscosity = 0.0035 poise). Pulsating velocity profiles from transcranial Doppler are imposed at the intake, and an open border with no dynamic pressure defines the outflow (20). Utilizing a time step of 0.001 s generates 800 solutions for 3 cardiac cycles. The last simulated cardiac cycle is utilized for post-processing hemodynamic parameters (4). Finally, morphological and hemodynamic outcomes are exported together.

### Clinical, morphological, and hemodynamic parameters of AChA aneurysms

Clinical features included gender, hypertension, hyperlipemia, smoking, alcohol, aneurysmal family history, earlier SAH, age, and body mass index (BMI). Two clinical research coordinators meticulously acquired data from the hospital information system, ensuring its accuracy through verification by a neurovascular interventionalist. Importantly, all personnel involved remained blinded to patients’ individual details.

Morphological parameters of aneurysms comprised the presence of a daughter sac, inflow angle, aspect ratio (AR), size ratio (SR), ellipticity index (EI), non-sphericity index (NSI), undulation index (UI), size, diameter of the parent vessel, surface area, and volume. An extensive description of morphological variables obtained for this study could be found in previous studies (21–23).

In this investigation, we meticulously recorded and computed four crucial hemodynamic parameters: normalized wall shear stress (NWSS), average oscillatory shear index (OSI_ave_), low shear area (LSA), and relative residence time (RRT). Comprehensive insights into the calculation methods can be gleaned from the seminal works of Liu et al. (4) and Retarekar et al. (24).

The identification of the aneurysm neck was executed by two neurosurgeons, both uninformed about the patient’s particulars. Any disparities in their assessments were expertly resolved through the intervention of a third neurosurgeon boasting two decades of experience in the neuro-interventional domain.

### Statistical analysis

Continuous variables were summarized as either median (interquartile range) or mean ± standard deviation, while categorical data were expressed as percentages. Normality tests were conducted on the continuous variables within the training set. Subsequently, our team explored group differences utilizing the Student’s *t*-test or Mann–Whitney U test for continuous variables and the chi-square test or Fisher’s exact test for categorical data. Logistic regression models, encompassing both univariate and multivariate analyses, were executed to identify independent variables associated with AChA aneurysm rupture. A backward stepwise selection approach was employed, where univariate analysis parameters with a significance level of *p* < 0.2 were incorporated into the multivariate regression models. Following the exclusion of parameters exhibiting a variance inflation factor (VIF) exceeding 4.0, those with the minimum Akaike information criterion (AIC) were selected to formulate nomogram models. Additionally, additive and multiplicative interaction analyses were performed on these factors during model construction. Model 1 integrated clinical, morphological, and hemodynamic (C + M + H) characteristics, while Model 2 included solely clinical and morphological (C + M) variables. Homogeneity between the training and external validation sets was assessed with a significance threshold set at *p* = 0.05.

The discriminative efficacy of the nomograms and the external validation set was elucidated through the associated area under the curve (AUC) and the receiver operating characteristic curve (ROC). Calibration plots illustrated the congruence between the predicted rupture and the observed outcomes. Moreover, the clinical utility of the model was substantiated via decision curve analysis (DCA), quantifying the model’s net benefit (higher values indicative of greater therapeutic efficacy) (25). Internal validation involved resampling with 1,000 bootstrap samples. The performance of model 1 was compared to that of model 2 in terms of AUC value, sensitivity, specificity, positive predictive value (PPV), negative predictive value (NPV), and accuracy. The DeLong test was employed to compare the AUC values of the two ROC curves. Additionally, integrated discrimination improvement (IDI) and net reclassification index (NRI) were computed to measure improvements in assessment and assess clinical utility.

A significance level of *p* < 0.05 (two-tailed) was deemed statistically significant in all conducted tests. Statistical analyses and graphical representations were conducted using R version 4.1.3.^1^ Adherence to the TRIPOD guidelines was observed in this research (26).

## Results

### Characteristics of patients and AChA aneurysms

In the study cohort, a total of 143 AChA aneurysms were initially included. Subsequently, 23 AChA aneurysms were excluded, resulting in a final analysis of 120 aneurysms. Among these, 54 were identified as ruptured, while 66 remained unruptured. It is noteworthy that the possibility of vasospasm in the parent vessel could not be ruled out in the four patients who experienced SAH lasting longer than 24 h. Additionally, nine aneurysms were deemed ineligible, comprising two distal AChA dissecting aneurysms and seven multiple aneurysms. Furthermore, 10 patients with inadequate data were excluded from measurement and calculation (Figure 1). The multidimensional data for both the training and test sets are detailed in Supplementary Table S1. Statistically significant differences (*p* < 0.05) between the two groups were observed in parameters such as BMI (*p* = 0.01), NWSS × 10 (*p* = 0.03), OSI_ave_ × 10 (*p* = 0.02), LSA (*p* = 0.01), and RRT (*p* = 0.01). Conversely, parameters such as gender (*p* = 0.70), hypertension (*p* = 0.52), hyperlipidemia (*p* = 0.37), smoking (*p* = 0.96), alcohol (*p* = 0.34), family history (*p* = 0.14), earlier SAH (*p* = 0.23), age (*p* = 0.37), AR (*p* = 0.01), presence of the daughter sac (*p* = 0.94), inflow angle (*p* = 0.44), SR (*p* = 0.30), EI × 10 (*p* = 0.10), NSI × 10 (*p* = 0.30), UI × 10 (*p* = 0.69), diameter of the parent vessel (*p* = 0.30), size (*p* = 0.50), surface area (*p* = 0.24), and volume (*p* = 0.18) did not exhibit statistically significant differences. Table 1 provides a detailed presentation of the results obtained from the Shapiro–Wilk and VIF tests conducted on ruptured and unruptured aneurysms within the training set.

**Table 1:** Clinical, morphological, and hemodynamic features of AChA aneurysms in the training set.

Features	Overall (*n* = 98)	Unruptured (*n* = 53)	Ruptured (*n* = 45)	Shapiro–Wilk (*p*-value)	VIF
Clinical features					
Gender (male), %	40 (40.82)	21 (39.62)	19 (42.22)	**/**	1.36
Hypertension (yes), %	65 (66.33)	33 (62.26)	32 (71.11)	**/**	1.85
Hyperlipemia (yes), %	11 (11.22)	6 (11.32)	5 (11.11)	**/**	1.23
Smoking (yes), %	13 (13.27)	3 (5.66)	10 (22.22)	**/**	1.56
Alcohol (yes), %	17 (17.35)	8 (15.09)	9 (20.00)	**/**	1.54
Family history (yes), %	9 (9.18)	7 (13.21)	2 (4.44)	**/**	1.34
Earlier SAH (yes), %	6 (6.12)	3 (5.66)	3 (6.67)	**/**	1.45
Age, years	57 (47, 64)	58 (45, 66)	57 (48, 63)	0.019	1.67
BMI, kg/m^2^	24.11 ± 2.01	24.02 ± 1.76	24.21 ± 2.26	0.168	2.32
Morphological features					
Daughter sac (yes), %	23 (23.47)	2 (3.77)	21 (46.67)	**/**	1.90
Inflow angle, °	95.50 ± 32.07	97.40 ± 35.35	93.25 ± 27.54	0.274	1.21
AR	1.14 (0.80, 1.54)	0.88 (0.71, 1.29)	1.34 (1.11, 1.77)	< 0.001	4.54
SR	1.13 (0.88, 1.53)	1.04 (0.74, 1.33)	1.29 (1.06, 1.74)	< 0.001	3.97
EI **×** 10	1.39 (0.64, 1.84)	0.77 (0.40, 1.48)	1.81 (1.56, 2.00)	0.001	31.67
NSI **×** 10	1.74 (0.82, 2.20)	0.94 (0.50, 1.72)	2.08 (1.91, 2.44)	0.005	28.04
UI **×** 10	0.73 (0.35, 1.20)	0.42 (0.24, 0.84)	1.00 (0.65, 1.51)	< 0.001	2.47
Size, mm	3.15 (2.20, 4.10)	2.49 (1.87, 3.38)	3.67 (3.11, 5.00)	0.004	3.98
Diameter of parent vessel, mm	2.71 ± 0.63	2.62 ± 0.62	2.80 ± 0.61	0.238	2.49
Surface area, mm^2^	24.99 (15.52, 38.41)	21.78 (13.33, 28.11)	29.16 (21.60, 40.50)	< 0.001	43.57
Volume, mm^3^	12.33 (6.64, 23.92)	9.27 (5.34, 15.12)	17.14 (9.33, 28.31)	< 0.001	3.90
Hemodynamic features					
NWSS **×** 10, Pa	2.83 (1.38, 5.78)	4.67 (2.18, 6.78)	1.95 (1.04, 4.08)	< 0.001	2.00
OSI_ave_ **×** 10	1.60 (0.90, 2.50)	1.40 (0.80, 2.10)	1.90 (1.40, 2.60)	< 0.001	1.38
LSA, %	23.15 (0.04, 62.86)	1.74 (0.02, 27.05)	49.55 (25.25, 79.67)	< 0.001	4.37
RRT, s	1.03 (0.36, 4.30)	0.45 (0.25, 1.03)	3.28 (1.21, 11.57)	< 0.001	2.36

Continuous variables were expressed in mean ± standard deviation (SD) or median (interquartile range). Categorical variables were expressed as number of patients (%). VIF, variance inflation factor; AChA, anterior choroidal artery; SAH, subarachnoid hemorrhage; AR, aspect ratio; SR, size ratio; EI, ellipticity index; NSI, non-sphericity index; UI, undulation index; NWSS, normalized wall shear stress; OSI_ave_, average oscillatory shear index; LSA, low wall shear stress area; RRT, relative residence time.

### Nomogram variable screening

Following univariate logistic regression analysis (*p* < 0.2), 16 variables were retained for further examination in the context of AChA aneurysm rupture assessment. These variables encompassed smoking, aneurysmal family history, the presence of a daughter sac, AR, SR, EI, NSI, UI, size, diameter of the parent vessel, surface area, volume, NWSS, OSI_ave_, LSA, and RRT. Assessment of VIF was conducted, with values exceeding 4.0 indicating multicollinearity. Variables surpassing this threshold were systematically excluded from the final model analysis, as outlined in Table 1. In the subsequent multivariate logistic regression analysis, employing backward stepwise selection combined with AIC minimization, smoking (*p* = 0.033, OR = 3.83), SR (*p* = 0.031, OR = 2.40), NWSS (*p* = 0.034, OR = 0.83), and OSI_ave_ (*p* = 0.301, OR = 1.10) emerged as independent predictors for AChA aneurysm rupture, as detailed in Table 2.

**Table 2:** Univariate and multivariate regression analyses of the training set.

Features	Univariate		Multivariate	
	OR (95% CI)	*P*-value	OR (95% CI)	*P*-value
Clinical features				
Gender (male vs. female), %	1.11 (0.50, 2.50)	0.794	**/**	**/**
Hypertension (yes vs. no), %	1.49 (0.64, 3.49)	0.357	**/**	**/**
Hyperlipemia (yes vs. no), %	1.48 (0.42, 5.21)	0.544	**/**	**/**
Smoking (yes vs. no), %	3.90 (1.27, 15.01)	0.017	3.83 (1.12, 13.11)	0.033
Alcohol (yes vs. no), %	1.41 (0.49, 4.01)	0.524	**/**	**/**
Family history (yes vs. no), %	0.31 (0.06, 1.55)	0.153	**/**	**/**
Earlier SAH (yes vs. no), %	1.19 (0.23, 6.21)	0.836	**/**	**/**
Age, years	0.99 (0.96, 1.03)	0.640	**/**	**/**
BMI, kg/m^2^	1.02 (0.98, 1.09)	0.657	**/**	**/**
Morphological features				
Daughter sac (yes vs. no), %	7.29 (1.51, 35.30)	0.014	**/**	**/**
Inflow angle, °	1.00 (0.98, 1.01)	0.535	**/**	**/**
AR	1.21 (1.09, 1.54)	<0.001	**/**	**/**
SR	3.01 (1.37, 6.59)	0.006	2.40 (1.08, 5.32)	0.031
EI **×** 10	1.25 (1.14, 1.36)	<0.001	**/**	**/**
NSI **×** 10	1.20 (1.12, 1.30)	<0.001	**/**	**/**
UI **×** 10	3.12 (1.55, 6.28)	0.011	**/**	**/**
Size, mm	2.29 (1.53, 3.41)	0.019	**/**	**/**
Diameter of parent vessel, mm	2.12 (1.09, 4.38)	0.022	**/**	**/**
Surface area, mm^2^	3.32 (1.45, 8.11)	0.031	**/**	**/**
Volume, mm^3^	5.69 (1.76, 12.51)	0.029	**/**	**/**
Hemodynamic features				
NWSS **×** 10, Pa	0.82 (0.70, 0.96)	0.013	0.83 (0.69, 0.99)	0.034
OSI_ave_ **×** 10	1.15 (0.95, 1.39)	0.085	1.10 (0.93, 1.28)	0.301
LSA, %	1.03 (1.02, 1.05)	0.003	**/**	**/**
RRT	1.01 (0.99, 1.03)	0.180	**/**	**/**

OR, odds ratio; SAH, subarachnoid hemorrhage; AR, aspect ratio; SR, size ratio; EI, ellipticity index; NSI, non-sphericity index; UI, undulation index; NWSS, normalized wall shear stress; OSIave, average oscillatory shear index; LSA, low wall shear stress area; RRT, relative residence time.

### Construction and performance of nomogram models

**Figure 2:**
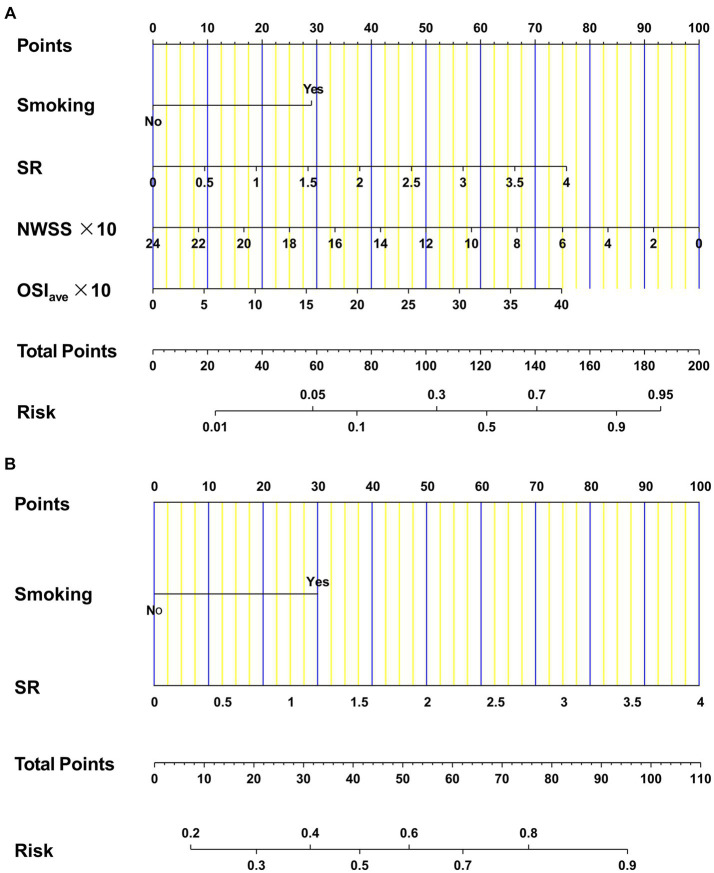
Nomograms for AChA aneurysms regarding clinical, morphological, and hemodynamic candidates. Model 1 **(A)** was constructed by four independent factors: smoking, SR, NWSS, and OSI_ave_. Model 2 **(B)** was developed by two independent factors: smoking and SR. AChA, anterior choroid artery; SR, size ratio; NWSS, normalized wall shear stress; OSI_ave_, average oscillatory shear index.

To assess the likelihood of rupture in AChA aneurysms, we developed two binary logistic regression models incorporating four independent risk variables. Model 1 was expressed as Log[p(An)/1 − p(An)] = 1.342 ∗ smoking +0.875 ∗ SR − 0.193 ∗ (NWSS * 10) + 0.086 ∗(OSI_ave_ * 10) − 1.029, where p(An) denotes the estimated rupture probability. The other model, denoted as model 2 (C + M), was represented as Log[p(An)/1 − p(An)] = 1.239 ∗ smoking +1.018 ∗ SR − 1.683. Figure 2 provides a detailed visualization of both models using multidimensional nomograms.

**Figure 3:**
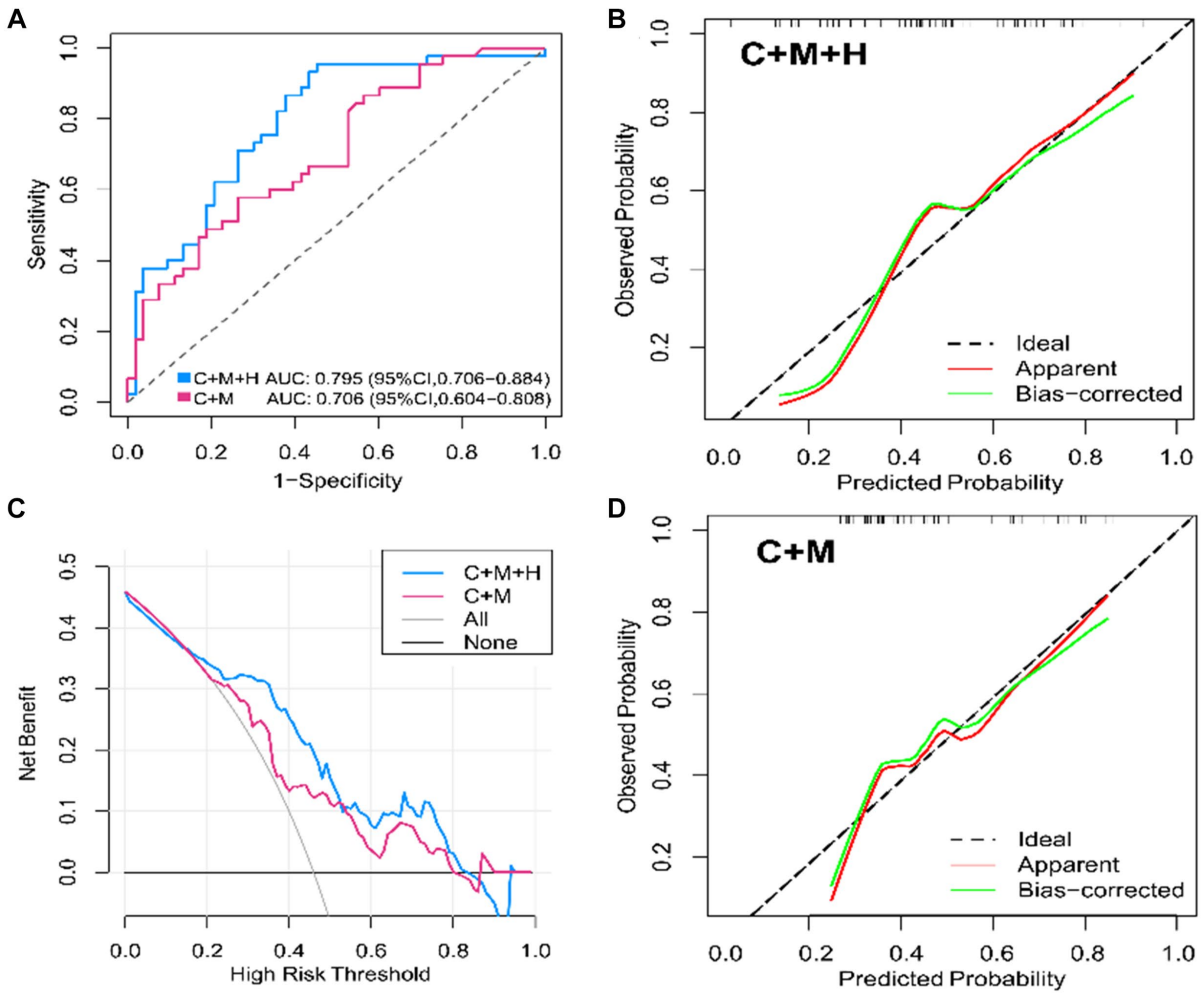
ROC curves, calibration plots, DCA for model 1 (C + M + H) and model 2 (C + M) in the training set. In models 1 (C + M + H) and 2 (C + M), the AUC values were 0.795 (95% CI, 0.706 ~ 0.884) and 0.706 (95% CI, 0.604 ~ 0.808), respectively. **(A)** The horizontal and vertical axes illustrate, respectively, the evaluated and observed probabilities of aneurysmal rupture. The dashed lines reveal an ideal model’s flawless prediction. The green lines show inner validation by 1,000 bootstrap resampling. The red lines demonstrate the performance of the nomogram model. **(B,C)** The horizontal and vertical axes represent the threshold for determining great risk and clinical net benefit, respectively. The blue and red lines represent the net benefits of Models 1 (C + M + H) and 2 (C + M), respectively. At Y = 0, the horizontal line represents no treatment, while the gray curve means thorough intervention. **(D)** ROC, receiver operator characteristic; DCA, decision curve analysis; C + M + H, clinical, morphological, and hemodynamic features; C + M, clinical and morphological features; AUC, area under curve; CI, confidence interval.

In the training dataset, both model 1 (AUC = 0.795; 95% CI, 0.706 ~ 0.884) and model 2 (AUC = 0.706; 95% CI, 0.604 ~ 0.808) demonstrated significant discriminatory capabilities (Figure 3A). Additionally, by setting the optimal cutoff at 0.346, model 1 (C + M + H) achieved sensitivity, specificity, PPV, and NPV values of 95.6, 54.7, 64.2, and 93.6%, respectively. Model 2 (C + M) exhibited corresponding values of 82.4, 50.3, 55.9, and 76.7%, respectively (Supplementary Table S2).

**Figure 4:**
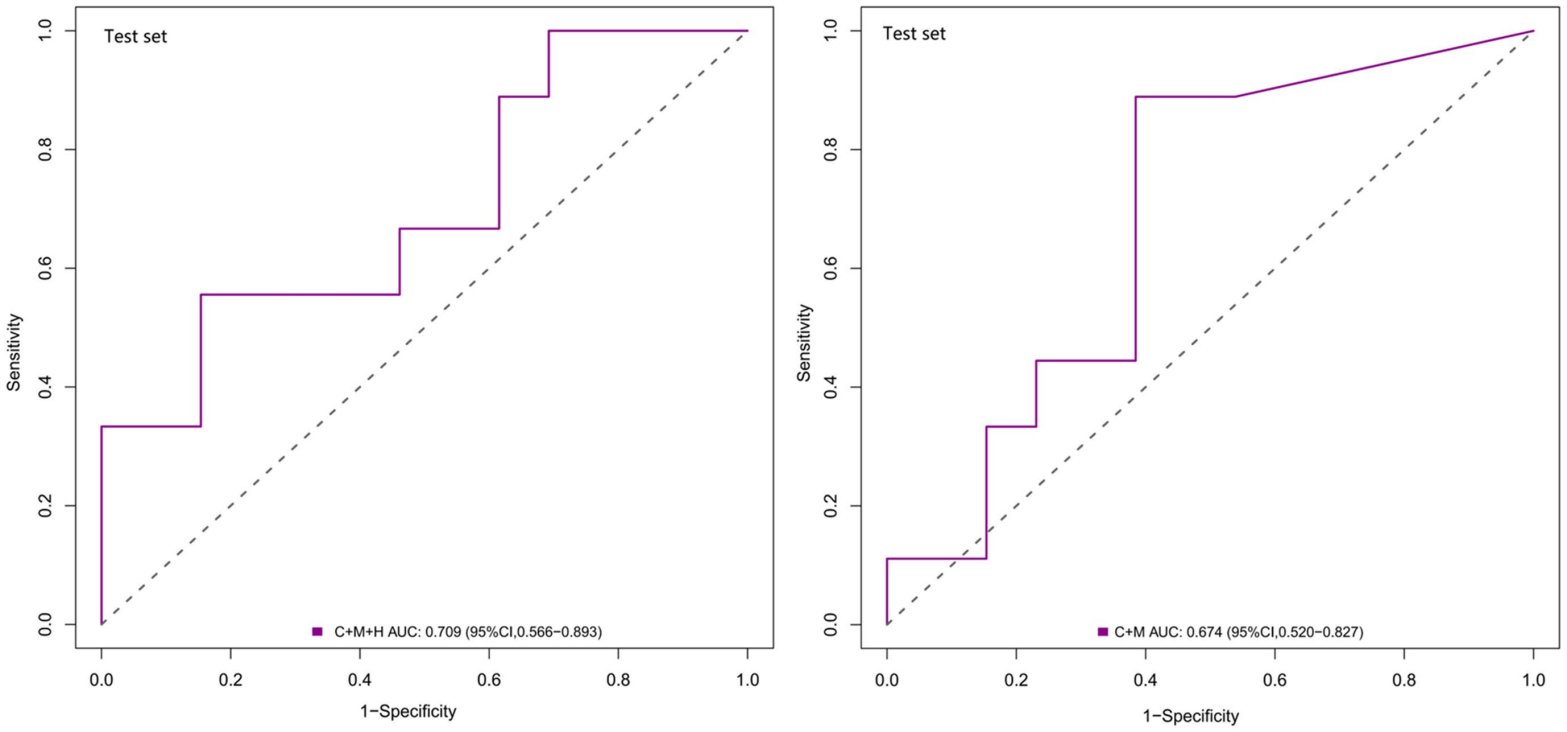
ROC curves for model 1 (C + M + H) and model 2 (C + M) in the validation set. The AUCs for model 1 (C + M + H) and model 2 (C + M) were 0.709 (95% CI: 0.566 ~ 0.893) and 0.674 (95% CI: 0.520 ~ 0.827), respectively. ROC, receiver operator characteristic; C + M + H, clinical, morphological, and hemodynamic features; C + M, clinical and morphological features; AUC, area under curve; CI, confidence interval.

Based on the observations in the training set, the external validation set revealed that model 1 (AUC = 0.709; 95% CI, 0.566 ~ 0.893) demonstrated superior discriminative capability than model 2 (AUC = 0.674; 95% CI, 0.520 ~ 0.827) (Figure 4). Moreover, utilizing 0.337 as the optimal cutoff value, model 1 (C + M + H) exhibited a sensitivity of 82.7%, specificity of 53.2%, NPV of 58.2%, and PPV of 80.5%. In contrast, model 2 (C + M) showed a sensitivity of 46.1%, specificity of 78.6%, NPV of 61.8%, and PPV of 64.7% (Supplementary Table S3).

**Figure 5:**
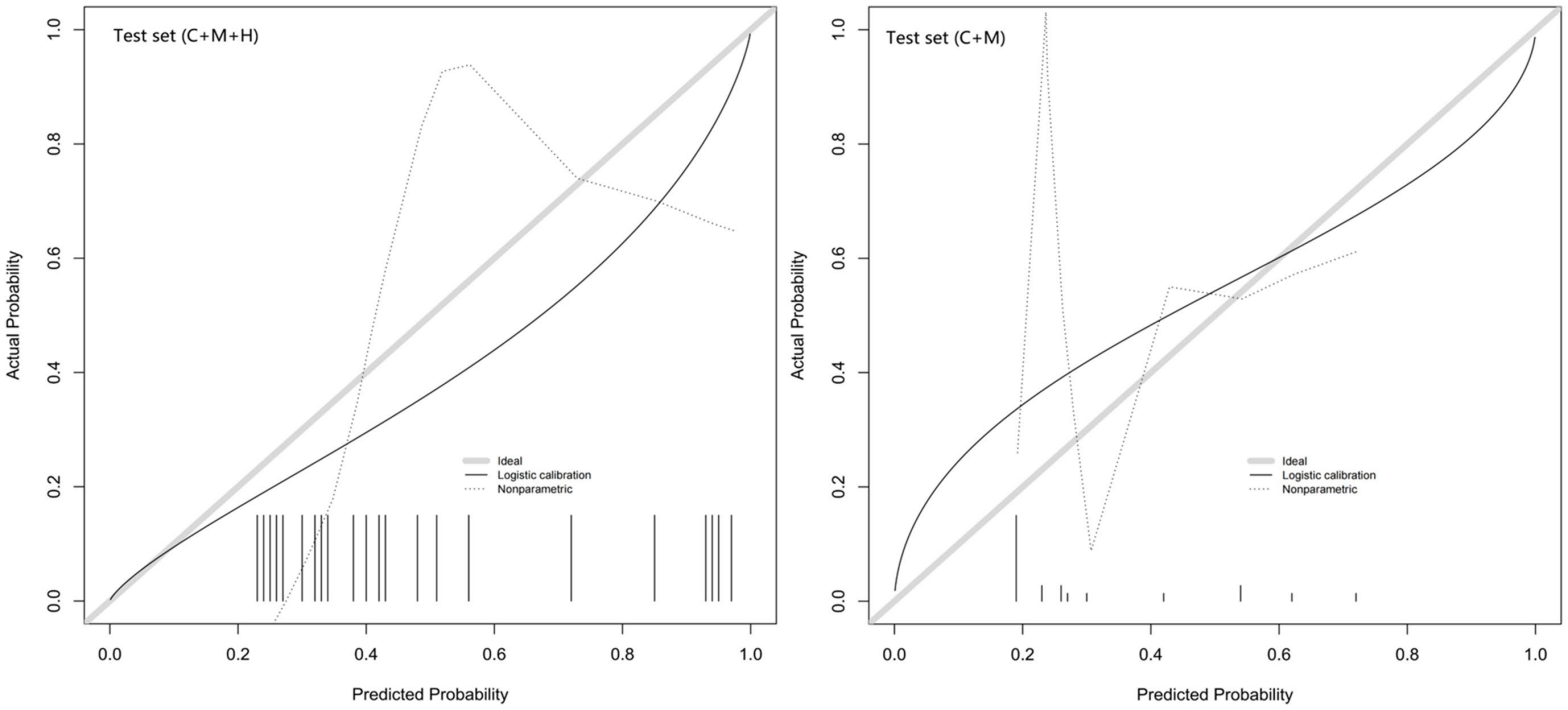
Calibration curves of model 1 (C + M + H) and model 2 (C + M) in the validation set. The calibration curve describes the calibration of the external validation set. The x-axis and y-axis represent the model estimated and actual values, respectively. The diagonal gray lines represent model-perfect assessments. The level of accuracy increases as the black line approaches the gray line. C + M + H, clinical, morphological, and hemodynamic features; C + M, clinical and morphological features.

**Figure 6:**
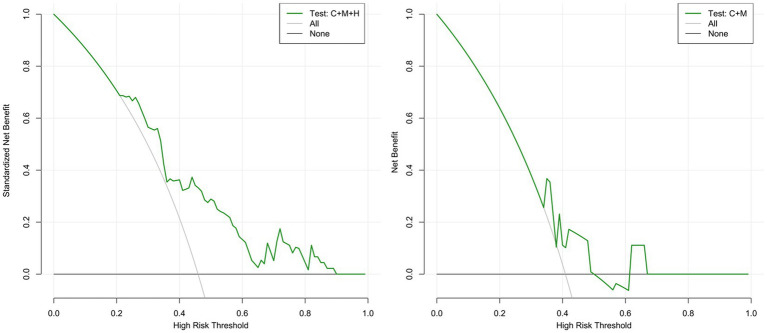
DCA curves for model 1 (C + M + H) and model 2 (C + M) in the validation set. The green lines represent the net benefits of models 1 (C + M + H) and 2 (C + M) in the validation set. The horizontal line (Y = 0) and the gray curve indicate no treatment (treat-none) and radical intervention (treat-all), respectively. DCA, decision curve analysis; C + M + H, clinical, morphological, and hemodynamic features; C + M, clinical and morphological features.

The calibration plots for 2 models were generated based on 1,000 iterations of bootstrap sampling. The results presented in Figures 3B,C, 5 illustrate a significant alignment between the observed state of instability and the predicted risk of rupture evaluated through nomograms. Notably, the calibration plots reveal a higher level of consistency in the external validation set than in the training set. Additionally, Figures 3D, 6 display DCA curves, indicating that model 1 (C + M + H) exhibited superior performance and was deemed suitable for guiding more advantageous clinical decisions.

### Clinical value of two multidimensional models

In comparison with model 2 (AUC = 0.706; 95% CI, 0.604 ~ 0.808), model 1 (AUC = 0.795; 95% CI, 0.706 ~ 0.884) demonstrated superior discriminatory ability in the training set, as evidenced by the DeLong test (change = 0.089, *p* = 0.008) (Figure 3A; Supplementary Table S4). The DeLong test was employed to contrast model 1 (AUC = 0.709; 95% CI, 0.566 ~ 0.893) with model 2 (AUC = 0.674; 95% CI, 0.520 ~ 0.827) in the external validation set. The determined model improvement value was 0.035 (95% CI, 0.007 ~ 0.114, *p* = 0.047) (Figure 4; Supplementary Table S5). Importantly, the changes in NRI and IDI were utilized to assess the efficacy of the nomogram models, with NRI values of 0.224 (95% CI, 0.06 ~ 0.388, *p* = 0.007) and 0.624 (95% CI, 0.168 ~ 1.107, *p* = 0.063) in the two cohorts, and IDI values of 0.572 (95% CI, 0.173 ~ 0.811, *p* = 0.044) and 0.585 (95% CI, 0.208 ~ 0.963, *p* = 0.002), respectively (Supplementary Tables S5, S6). Overall, the above-presented results indicate that model 1, incorporating hemodynamic features, may effectively discern the rupture status of AChA aneurysms compared to model 2. Additionally, DCA curves reveal that model 1 (C + M + H) yields greater net benefits than model 2 (C + M) under both treat-all and treat-none strategies (Figures 3D, 6).

## Discussion

Rupture risk assessment of AChA aneurysms plays a pivotal role in the medical management of those yet to rupture. Through a training set encompassing 98 cases, we identified 4 contributors (smoking, SR, NWSS, and OSI_ave_) for constructing nomogram models. Subsequently, two models were devised and juxtaposed to discern the rupture probability of AChA aneurysms. Model 1 (C + M + H) showcased superior performance in both training and validation sets, excelling in discrimination, calibration, and clinical applicability in contrast to model 2 (C + M). The clinical and morphological parameters in model 2 (C + M) were conveniently accessible, enabling interventionalists to swiftly make initial assessments. The precision of model 1 (C + M + H) and the accessibility of model 2(C + M) might yield distinct advantages.

In this study, clinical features independent of morphological and hemodynamic factors were identified as significant determinants of aneurysm rupture (27). Specifically, smoking emerged as a distinct risk factor for AChA aneurysm rupture. Notably, Juvela (28) integrated smoking into the PHASES rating scale, enhancing its efficacy in assessing long-term rupture risks in UIAs. Consistent with our findings, a case–control analysis of 4,701 individuals with 6,411 aneurysms demonstrated that smokers faced a heightened risk of rupture compared to non-smokers (29). Additionally, while hypertension (30), earlier SAH (11), and familial history of aneurysm (31) were commonly recognized as risk factors for UIAs, our study did not find these variables to be statistically significant, potentially due to the limitations of our sample size.

Notably, morphological parameters of aneurysms have been empirically linked to their propensity for rupture (32). SR encompasses the combined influence of the aneurysm and adjacent arteries, denoting the ratio of aneurysmal height to the average diameter of the parent artery (21). A substantial cohort, comprising 854 ruptured aneurysms and 180 UIAs, divulged that SR, rather than aneurysmal size, plays a pivotal role in predicting rupture status, especially in small aneurysms (<5 mm) (33). Furthermore, the investigations by Xiang et al. (34) and Yuan et al. (35) supported our findings, revealing a robust correlation between high SR and aneurysmal rupture. Interestingly, previous studies observed disparate cutoff values for SR, potentially attributable to variations in study design, particularly single-site methodologies. Our study evidenced that the ruptured group exhibited higher values for AR, EI, NSI, UI, size, and the presence of a daughter sac than the unruptured group, aligning with previous research (32). Despite SR being the sole morphological feature integrated into our models, it does not negate the significance of the seven other contributors in the rupture of AChA aneurysms.

The morphology and hemodynamics of aneurysms are intricately connected, with poor morphology exacerbating the process of aneurysmal growth and rupture by influencing hemodynamics (34). The impact of wall shear stress (WSS) on aneurysm growth and rupture remains a subject of debate. In a retrospective analysis of 210 aneurysms, it was suggested that ruptured aneurysms exhibit an increased WSS (36). In contrast, Xiang et al. (34) and Leemans et al. (37), through morphological measurement and hemodynamic calculation of cerebral aneurysms at various sites, found that a lower WSS serves as an independent predictor of aneurysmal rupture. Variations in the anatomical characteristics of aneurysms at different locations may influence the hemodynamic pattern (38). Therefore, it is advisable to conduct hemodynamic evaluations on aneurysms in a single location for prudence. Both Duan et al. (19) and Yuan et al. (35) discovered an association between decreased WSS and rupture status in posterior communicating artery (PCoA) aneurysms. Extremely low WSS could prompt the endothelium to interface with adhesive molecules, activate proinflammatory cytokines, and inevitably lead to lesion wall deterioration and rupture (39). These site-specific investigations underscore the significance of low WSS in aneurysm rupture.

Alongside WSS, OSI_ave_ emerges as another pertinent hemodynamic parameter in aneurysm rupture dynamics (34). Despite our findings indicating non-statistical significance, OSI_ave_ found its place in the final models based on AIC minimum criteria. In a study focusing on PCoA aneurysms, OSIave demonstrated higher values in ruptured aneurysms than in unruptured counterparts, albeit lacking statistical significance (*p* > 0.05) (40). The patterns observed in AChA aneurysms mirror those in PCoA. Given our constrained sample size, leveraging extensive datasets for robust validation remains imperative in future investigations.

Similar to our findings, a retrospective study involving 119 IAs found that high SR, low WSS, and high OSI_ave_ were risk factors for the rupture of IAs. The derived regression equations yielded an AUC of 0.89 for the comprehensive model, incorporating hemodynamic parameters, marking the highest predictive accuracy (41). Moreover, Liu et al. (4) categorized the rupture risk concerning the stability of IAs in the anterior circulation. The C-indices for the models (C + M + H), (C + M), and PHASES scores were 0.94, 0.89, and 0.68, respectively. Our current investigation identified similar risk factors as previous studies but exhibited variable efficacy, potentially attributable to the exclusive focus of our models on AChA aneurysms.

Machine learning has played a pivotal role in the extensive identification and assessment of rupture risk in IAs. Through a comprehensive analysis of various algorithms, Ou et al. (14) and Xiong et al. (15) have substantiated that the predictive accuracy of machine learning significantly surpasses that of logistic regression models and scoring systems. Conversely, a multicenter study conducted in China revealed that traditional logistic regression is not inferior to machine learning algorithms in multidimensional models for predicting the rupture status of unruptured IAs (42). Therefore, it is essential to undertake a comparative analysis of several models for AChA aneurysms and carefully select the optimal model for subsequent external validation. This approach is indispensable for fostering continual improvement in predicting the rupture risk of AChA aneurysms in the future.

## Limitations

In conducting this preliminary exploratory study on the assessment of AChA aneurysm risk, several limitations persist. First, the study is retrospective, and future endeavors should involve the advancement of predictive models through a multicenter prospective cohort to enable personalized evaluation of the rupture risk associated with AChA aneurysms. Second, the occurrence of instantaneous morphological and hemodynamic changes before aneurysm rupture poses a formidable challenge in capturing these changes at their pre-ruptured state in clinical practice. Additionally, a previous study indicated that morphological changes before and after the rupture of the majority of IAs were not statistically significant (43). Third, the absence of patient-specific data for CFD simulation with the software may hinder the widespread application of assessment models in real-world scenarios. Fourth, the small sample size could introduce selection bias and result in low specificity. Although there were 98 cases in the internal training set and 22 in the external test set, representing considerable numbers for AChA aneurysms, the sample sizes remained statistically small. Finally, the employed statistical methodologies lacked novelty, and machine learning algorithms were not incorporated. Collaborative efforts with artificial intelligence engineers are underway to develop interpretable algorithms suitable for application to small datasets.

## Conclusion

We have developed and validated two assessment models to evaluate the risk of rupture in AChA aneurysms. Model 1 (C + M + H) exhibited superior accuracy, calibration, and clinical utility, whereas model 2 (C + M) possessed the advantage of time efficiency. These nomogram models represent valuable tools for conducting personalized risk assessments of unruptured AChA aneurysms.

## Data availability statement

The original contributions presented in the study are included in the article/Supplementary material, further inquiries can be directed to the corresponding authors.

## Ethics statement

The studies involving humans were approved by the Shanghai Changhai Hospital Ethics Committee Board. The studies were conducted in accordance with the local legislation and institutional requirements. The ethics committee/institutional review board waived the requirement of written informed consent for participation from the participants or the participants' legal guardians/next of due to the retrospective nature of the study.

## Author contributions

SZ: Writing – original draft, Writing – review & editing, Data curation, Formal analysis, Software. XX: Writing – original draft, Writing – review & editing, Formal analysis, Investigation. RZ: Data curation, Formal analysis, Methodology, Software, Writing – original draft. ZL: Data curation, Investigation, Writing – original draft. YY: Data curation, Formal analysis, Writing – review & editing. SL: Data curation, Investigation, Writing – original draft. YW: Data curation, Investigation, Writing – original draft. JC: Data curation, Investigation, Writing – original draft. LL: Data curation, Investigation, Writing – original draft. JX: Conceptualization, Funding acquisition, Project administration, Supervision, Writing – review & editing. QH: Conceptualization, Funding acquisition, Project administration, Supervision, Writing – review & editing.

## Footnotes

^1^ https://www.r-project.org/
