# Supplementary material for: Nomograms for assessing the rupture risk of anterior choroid artery aneurysms based on clinical, morphological, and hemodynamic features
Source: Front Neurol. 2024 Feb 8;15:1304270. doi: 10.3389/fneur.2024.1304270 (PMC10882079; doi:10.3389/fneur.2024.1304270)
Supplement: Supplementary file 1 [file Table_1.DOCX]

**Supplementary table 1. Comparison of training and validation sets**

| Features | Overall (n = 120) | Training set (n = 98) | Validation set (n = 22) | P value |
| --- | --- | --- | --- | --- |
| **Clinical features** |  |  |  |  |
| Gender(male), % | 48 (40.0) | 40 (40.8) | 8 ( 36.4) | 0.70 |
| Hypertension(yes), % | 78 (65.0) | 65 (66.3) | 13 ( 59.1) | 0.52 |
| Hyperlipemia(yes), % | 15 (12.5) | 11 (11.2) | 4 (18.2) | 0.37 |
| Smoking(yes), % | 16 (13.3) | 13 (13.3) | 3 ( 13.6) | 0.96 |
| Alcohol(yes), % | 19 (15.8) | 17 (17.3) | 2 (9.1) | 0.34 |
| Family history(yes), % | 9 ( 7.5) | 9 ( 9.2) | 0 (0.0) | 0.14 |
| Earlier SAH(yes), % | 6 ( 5.0) | 6 ( 6.1) | 0 (0.0) | 0.23 |
| Age, years | 57.50 (48.00, 64.00) | 57.00 (47.25, 64.00) | 59.00 (51.50, 64.25) | 0.37 |
| BMI, kg/m^2^ | 24.09 ± 1.98 | 23.98 ± 1.80 | 25.96 ± 2.64 | 0.01 |
| **Morphological features** |  |  |  |  |
| Daughter sac(yes), % | 28 (23.3) | 23 (23.5) | 5 (22.7) | 0.94 |
| Inflow angle, **°** | 96.32 ± 31.04 | 95.08 ± 36.49 | 107.16 ± 23.12 | 0.44 |
| AR | 1.08 (0.80, 1.47) | 1.15 (0.80, 1.54) | 0.85 (0.66, 1.06) | 0.01 |
| SR | 1.14 (0.88, 1.55) | 1.13 (0.88, 1.53) | 1.30 (0.90, 2.26) | 0.30 |
| EI **×**10 | 1.31 (0.58, 1.81) | 1.40 (0.64, 1.84) | 0.92 (0.45, 1.60) | 0.10 |
| NSI **×**10 | 1.72 (0.70, 2.20) | 1.75 (0.82, 2.20) | 1.14 (0.62, 2.17) | 0.30 |
| UI ×10 | 0.72 (0.34, 1.21) | 0.75 (0.36, 1.19) | 0.60 (0.30, 1.28) | 0.69 |
| Size, mm | 3.18 (2.20, 4.21) | 3.16 (2.22, 4.08) | 3.37 (2.16, 5.03) | 0.50 |
| Diameter of parent vessel, mm | 2.64 (2.23, 3.07) | 2.68 (2.27, 3.06) | 2.52 (1.87, 3.11) | 0.30 |
| Surface area, mm^2^ | 25.36 (15.48, 44.28) | 25.02 (15.63, 38.24) | 33.50 (13.71, 64.10) | 0.24 |
| Volume, mm^3^ | 12.94 (6.62, 27.54) | 12.47 (6.67, 23.80) | 20.69 (6.17, 58.29) | 0.18 |
| **Hemodynamic features** |  |  |  |  |
| NWSS**×**10, Pa | 3.90 (1.85, 6.37) | 2.97 (1.40, 5.77) | 6.12 (3.92, 8.09) | 0.03 |
| OSI_ave_ **×**10 | 0.16 (0.08, 0.24) | 0.16 (0.10, 0.25) | 0.08 (0.03, 0.15) | 0.02 |
| LSA, % | 16.74 (0.00, 51.61) | 23.37 (0.00, 61.94) | 0.35 (0.00, 12.40) | 0.01 |
| RRT, s | 0.85 (0.32, 3.29) | 1.04 (0.36, 4.22) | 0.35 (0.21, 0.88) | 0.01 |

Continuous variables were expressed in mean ± standard deviation (SD) or median (interquartile range). Categorical variables were expressed as number of patients (%).

SAH, subarachnoid hemorrhage; AR, aspect ratio; SR, size ratio; EI, ellipticity index; NSI, nonspericity index; UI, undulation index; NWSS, normalized wall shear stress; OSI_ave_, average oscillatory shear index; LSA, low wall shear stress area; RRT, relative residence time.

**Supplementary table 2. Sensitivity, specificity, PPV, and NPV at the optimal cut-off value in the training set**

| Model | cut-off | Sensitivity(%) | Specificity(%) | PPV(%) | NPV(%) | Accuracy |
| --- | --- | --- | --- | --- | --- | --- |
| Model 1 | 0.346 | 95.6 | 54.7 | 64.2 | 93.6 | 0.74 (0.64 ~ 0.82) |
| Model 2 | 0.346 | 82.4 | 50.3 | 55.9 | 76.7 | 0.62 (0.52 ~ 0.72) |

PPV, positive predictive value; NPV, negative predictive value.

**Supplementary table 3. Sensitivity, specificity, PPV, and NPV at the optimal cut-off value in the validation set**

| Model | cut-off | Sensitivity(%) | Specificity(%) | PPV(%) | NPV(%) | Accuracy |
| --- | --- | --- | --- | --- | --- | --- |
| Model 1 | 0.336 | 82.7 | 53.2 | 58.2 | 80.6 | 0.66 (0.48 ~ 0.84) |
| Model 2 | 0.336 | 46.1 | 78.6 | 61.8 | 64.7 | 0.61 (0.33 ~ 0.91) |

PPV, positive predictive value; NPV, negative predictive value.

**Supplementary table 4. NRI, IDI, and AUC of the training set**

| **Training set** | **Estimate** | **95% CI** | **P value** |
| --- | --- | --- | --- |
| NRI (C+M+H vs. C+M) | 0.224 | (0.061-0.388) | **0.007** |
| IDI (C+M+H vs. C+M) | 0.585 | (0.208-0.963) | **0.002** |
| AUC(DeLong test for 2 ROC - curves) |  |  |  |
| Model 1 (C+M+H) | 0.795 | (0.706-0.884) | / |
| Model 2 (C+M) | 0.706 | (0.604-0.808) | / |
| AUC(DeLong test for 2 ROC - curves) | 0.089 | (0.024-0.155) | **0.008** |

CI, confidence interval; NRI, net reclassification index; IDI, integrated discrimination improvement; AUC, area under curve; AChA, anterior choroidal artery; C+M+H, clinical, morphological, and hemodynamic features; C+M, clinical and morphological features.

**Supplementary table 5. NRI, IDI, and AUC of the validation set**

|  | **Estimate** | **95% CI** | **P value** |
| --- | --- | --- | --- |
| NRI (C+M+H vs. C+M) | 0.624 | 0.168 ~ 1.107 | 0.063 |
| IDI (C+M+H vs. C+M) | 0.572 | 0.173 ~ 0.811 | **0.044** |
| AUC(DeLong test for 2 ROC - curves) |  |  |  |
| Model 1 (C+M+H) | 0.709 | 0.566 ~ 0.893 | / |
| Model 2 (C+M) | 0.674 | 0.520 ~ 0.827 | / |
| Change | 0.035 | 0.007 ~ 0.114 | **0.047** |

CI, confidence interval; NRI, net reclassification index; IDI, integrated discrimination improvement; AUC, area under curve; C+M+H, clinical, morphological, and hemodynamic features; C+M, clinical and morphological features.
